# A Perspective Of Intestinal Immune-Microbiome Interactions In Alcohol-Associated Liver Disease

**DOI:** 10.7150/ijbs.53589

**Published:** 2021-01-01

**Authors:** Ryan Bruellman, Cristina Llorente

**Affiliations:** Department of Medicine, University of California San Diego, La Jolla, CA, USA.

**Keywords:** Liver, intestine, microbiome, immune system, alcohol-associated liver disease

## Abstract

Uncovering the intricacies of the gut microbiome and how it interacts with the host immune system has opened up pathways in the search for the treatment of disease conditions. Alcohol-associated liver disease is a major cause of death worldwide. Research has shed light on the breakdown of the protective gut barriers, translocation of gut microbes to the liver and inflammatory immune response to microbes all contributing to alcohol-associated liver disease. This knowledge has opened up avenues for alternative therapies to alleviate alcohol-associated liver disease based on the interaction of the commensal gut microbiome as a key player in the regulation of the immune response. This review describes the relevance of the intestinal immune system, the gut microbiota, and specialized and non-specialized intestinal cells in the regulation of intestinal homeostasis. It also reflects how these components are altered during alcohol-associated liver disease and discusses new approaches for potential future therapies in alcohol-associated liver disease.

## Introduction

The significance and intricacy of the intestinal immune system cannot be underscored. Specialized cells within the intestine working with lamina propria immune cells are vital for protection against invading pathogens. The gut microbiota, comprising many different bacterial species is a major influencer on the intestinal immune system and is a necessary cog for proper intestinal immune function. Research studies continue to uncover new knowledge about the functionality of specialized cells within the intestine and how they interact with the gut microbiome and the immune system. This has allowed for further understanding to search for potential targets against conditions that are exacerbated due to dysfunctional intestinal homeostasis comprising a disrupted intestinal immune system and an altered intestinal microbiota such as the different stages of alcohol-associated liver disease (ALD). These different stages include steatosis, steatohepatitis, fibrosis, alcoholic hepatitis (AH), alcohol-associated cirrhosis (AC), and hepatocellular carcinoma. Steatosis in ALD is marked by the presence of lipids accumulating in the hepatocytes within the liver due to activation of lipogenic pathways, inhibition of fatty acid oxidation, alteration of very-low-density lipoprotein (VLDL) secretion from the liver, and alteration of triglycerides metabolism which accumulates in the form of lipid droplets 1, 2. Steatohepatitis also is marked by an increase in lipid content of the liver; however, inflammation is also evident with the increased abundance of inflammatory infiltrates consisting primarily of polymorphonuclear leukocytes (neutrophils) within the liver tissue 1, 2. Continuous damage to the liver through the fat deposits and inflammation causes the formation of scar tissue known as fibrosis, which over time and continual buildup can lead to cirrhosis of the liver 2. Long-term damage due to cirrhosis and inflammation of the liver also puts affected individuals at higher risk of developing hepatocellular carcinoma 1, 2. AH is a clinical diagnosis made due to chronic alcohol abuse paired with clinical findings 1. Hepatitis C antibodies have been found in a substantial amount of ALD patients with chronic alcohol abuse, and can further exacerbate ALD through inflammation and cirrhosis 3. In this review, we will discuss the major components that regulate the intestinal immune system. We will also discuss how these components are influenced by the gut microbiome and in particular, the changes that occur during ALD and its numerous stages. Finally, we will look into how the comprehensive understanding of the intestinal immune system has allowed for research into alternative therapies to attenuate ALD.

## Interactions of the intestinal epithelium and the intestinal immune system

The physical barriers of the intestine offer several lines of defense from potentially harmful pathogens as well as luminal contents and commensal bacteria from infiltrating the body under normal healthy conditions. Each barrier offers a distinctive structure that collectively functions to maintain a balance for both a healthy host as well as a healthy gut microbiome known as intestinal homeostasis. The intestinal epithelium, the innermost layer of the mucosa, is one of three major physical barriers that include specialized cells such as enterocytes, goblet cells, intestinal microfold (M) cells, enteroendocrine cells, Tuft cells, stem cells, and Paneth cells which are located in the crypts.

Enterocytes are present in large numbers in the small intestine but are also found in the large intestine, providing surveillance for protection 4. Enterocytes both secrete and respond to cytokines 5, 6. For instance, cytokines such as the proliferation-inducing ligand (APRIL) and the B cell-activating factor of the tumor necrosis factor family (BAFF) impact B cells and differentiated plasma cells while the thymic stromal lymphopoietin (TSLP) can impact dendritic cells (DCs) responses 7. In addition to cytokines, enterocytes also secrete antimicrobial peptides (AMPs) such as ß-defensins and regenerating islet-derived 3 (REG3) lectins that destroy bacteria by weakening the cell membrane through the formation of specific pores 8. Alcohol induces downregulation of the host antimicrobial proteins REG3ß and REG3γ in humans and mice 9-11. Cathelicidins are another type of AMPs secreted by the enterocytes in the small and large intestine and can disrupt cell membranes of bacteria and fungi in a similarly to REG3 AMPs 12. The secretion of AMPs is driven by toll-like receptors (TLRs) such as TLR4 and nucleotide-binding oligomerization domain-containing proteins (NODs) such as NOD2 along with the cells of the intestinal epithelial layer 13. In addition, enterocytes assist with adaptive immune responses by facilitating immunoglobulin A (IgA) transport across the epithelial barrier to the lumen through the transcytosis using the polymeric immunoglobulin receptor (pIgR) 14-16. IgA is an essential component in preventing microbe invasion across the intestinal barriers from the lumen 17. These offer a direct response and method of defense to invading microorganisms. Enterocytes also play a large role in controlling inflammatory immune responses as a major barrier from microbial invasions with the help of tight junctions between the epithelial cells and the proteins that it sheds such as Ly6/Plaur domain-containing 8 (Lypd8) 18. For instance, Lypd8 has been identified as a protein that helps to separate the intestinal microbiota and the epithelial tissue within the large intestine through binding to the flagella of certain bacteria 18. During ALD the enterocyte barrier of protection and the immune response is highly compromised 19-23. Ethanol-induced liver disease has been linked to increased levels of nitroxidative stress and altered tight junction proteins in mice, leading to increased intestinal permeability and bacteria crossing past the impaired barrier 24. Apoptosis of enterocytes has been documented in mice with ethanol-induced liver disease as noted by the increased protein-level expression of apoptosis markers further exacerbating the intestinal permeability condition and liver damage 24. In addition, fucosylated carbohydrate structures in intestinal epithelial cells play an important role in physiological and pathological processes 25-27. Among them, intestinal α1-2-fucosylation plays an important role in the regulation of intestinal homeostasis. Certain bacteria possess specific enzymes to hydrolyze α1-2-fucosidic linkages resulting in cleaved L-fucose, which facilitates bacterial colonization 28, 29. Fut2 is an enzyme that catalyzes the reaction of α1-2-fucosylation and is expressed in epithelial cells of the digestive tract while absent in the liver 30. Absence of α1-2-fucosylation along the cell surface of enterocytes and mucus results in alterations in intestinal bacteria, barrier function, and pathogen adhesion 25, 31. In duodenal biopsied of alcohol use disorder patients (AUD), α1-2-fucosylation is down-regulated, and the lack of α1-2-fucosylation in Fut2^-/-^ mice exacerbates ethanol-induced liver injury, steatosis, inflammation, and induces expansion of intestinal *Enterococcus faecalis (E. faecalis)* that produce a pore-forming toxin called cytolysin 25*.* These results suggest that modulation of intestinal α1-2-fucosylation could be used as a therapeutic approach to prevent ALDs.

Intestinal stem cells (ISCs) are an important structural and functional component of the intestinal immune system due to the ability to replenish themselves along with replenishing intestinal epithelial cells which can also differentiate to other specialized intestinal cell types 32. IL-6 coreceptor glycoprotein 130 (gp130) plays a key regulatory role in the regeneration and tissue growth of the intestine by activation of the gp130-YAP-Notch pathway 33. YAP expression also has been shown to impact Wnt/ß-catenin signaling and is typically upregulated during times of epithelial regeneration 34. Regeneration of intestinal epithelial cells is crucial for maintaining the barrier against invading microbes. Ethanol-induced liver disease damages ISCs through dysregulation of the ß-catenin signaling, a regulator of leucine-rich repeat-containing G-protein coupled receptor 5 (Lgr5) and other ISC markers in the small intestine of mice 32. This effectively stymies proper epithelial regeneration in the intestine during alcohol-induced liver disease, leading to increased bacterial translocation across the compromised intestinal barrier 32.

Paneth cells offer an imposing barrier to microbial products trying to pass the intestinal epithelial layer. Located predominately in the crypts of the small intestine adjacent to ISC in the small intestine, some Paneth cells can also be found in the proximal portion of the large intestine; however, during disease conditions, Paneth cells have been found in the distal portion of the large intestine 35. Paneth cells hold and secrete large amounts of AMPs to prevent microbial invasion. These AMPs are found throughout the mucus layer and offer an important component of protection from bacteria crossing the intestinal barrier due to their ability to kill microbes 36-39. Such AMPs include lysozyme C, α-defensins, phospholipases, cryptdins, and lectins. Lysozyme C is a noted AMP for preventing the buildup of peptidoglycans in tissues 40, 41. α-defensins, found in human Paneth cells, have similar functions to the ß-defensins. Cryptdins, which are found in mice, can also be referenced to α-defensins 42. Cryptdins can also be referenced to α-defensins, however, cryptdins are found in mice 42. Phospholipases from Paneth cells, which include secretory group IIA phospholipase A2 target gram-positive bacteria, target specific bacterial membrane components 43. Lectin AMPs known as REG3 are specifically noted for their ability to target and destroy harmful bacteria by damaging the cell wall 44. In mice, several different subsets of REG3 AMPs exist, including REG3α, REG3ß, and REG3γ. In humans REG3ß is not found, REG3α shares homology to mouse REG3γ, and REG3γ has considerable homology to mouse REG3ß 45. While each of these REG3 subsets targets gram-positive bacteria, each has distinct functions in immune responses. REG3α can penetrate gram-positive bacterial cell membranes via a hexameric membrane-penetrating pore formed through the binding of REG3α to a peptidoglycan 8. REG3ß and REG3γ both increase as a response to immune system activation by infection. REG3ß can also act to destroy gram-negative bacteria through the binding to carbohydrate portions of lipopolysaccharides on gram-negative bacteria 8. REG3γ helps to prevent bacteria from spreading on the epithelium along with mediating the spatial distance of the host from commensal microbes 8, 46. During ethanol-induced liver disease, Paneth cell function is altered. Chronic-binge ethanol feeding in mice has been tied to an increase in proximal small intestine Paneth cells along with IL-17A in mice, promoting an innate inflammatory immune system response 47. On the other hand, AMPs such as α-defensins were found to be reduced in male mice fed for 8 weeks ethanol-containing Lieber-DeCarli liquid diet 48. REG3 expression levels were found to be reduced in patients with ALD 10 which has been associated with an overall increase of mucosa-associated bacteria in the small intestine compared to the non-ALD controls 11. Expression of REG3 lectins was also reduced in mice after intragastric ethanol feeding 49, 50 as well as the ten-day feeding plus ethanol binge model 10 leading to a higher incidence of bacterial infiltration and translocation to the liver. In addition, REG3 knockout mice fed a long-term ethanol diet were found to have a higher liver injury due to more pronounced bacterial translocation 11.

Goblet cells also distribute AMPs such as resistin-like molecule ß (RELM-ß) and trefoil factor (TFF). RELM-ß acts to promote inflammation and immune response to invading pathogens through its ability to increase interferon-gamma (IFN-γ) expression in CD4 T cells 51. TFF2 is crucial due to its ability to help with epithelial repair and replacement in areas subject to mucosal damage 52. Goblet cells secrete an important component, mucin; that constitutes another major physical barrier known as the mucus layer, offering protection against luminal pathogens 53. The mucus layer is found throughout the digestive tract including the stomach, the small intestine, and the colon; however, the mucus has distinct properties in each section of the tract 54. MUC5AC is secreted by goblet cells to form the mucus layer in the stomach, whereas MUC2 is the major secreted component of the mucus layer throughout the intestine 54. This mucus layer serves as a buffer between gut microbes passing into the intestinal epithelium in both the small and large intestine 54. The mucus layer comprises an outer layer that has direct contact with the lumen and an inner layer which has direct contact with the intestinal epithelium. While mucin can keep most microbes from penetrating the barrier, other components of the mucus layer helps in the defense against these microbes. Goblet cells release RELM-ß into the mucus layer to destroy microbes that can penetrate the layer 55. On the inner areas of the mucus layer, AMPs derived from the Paneth cells and IgA, both prevent microbial adherence to the gut epithelium 46. Another major function of goblet cells includes the creation of goblet cell associated-antigen passages (GAPs) upon mucin secretion. GAPs within the small intestine are open and the GAPs in the colon remain closed to prevent an influx of excessive amounts of bacteria 56, 57. These GAPs play a crucial role in intestinal immune response as they send soluble luminal antigens to lamina propria dendritic cells (LP-DCs) 56. Once GAPs transport antigens to the lamina propria, LP-DCs provide an appropriate immune response. Antigen delivery of GAPs favors a certain subset of tolerogenic LP-DCs that are CD103+ 56. In mice with ethanol-induced liver disease promoted by the continuous intragastric feeding of an ethanol diet, mucin production in the intestinal system increases considerably, seemingly as a positive response to protect the intestine from bacterial invasion from the lumen 49. However, this thickening of the mucus layer is detrimental as AMPs struggle to control bacterial overgrowth in the lumen due to the wider physical barrier of the mucus layer between the intestinal epithelium and the lumen 49. Alcohol and its oxidative metabolites, specifically acetaldehyde can cause major changes in the intestinal barrier, increase paracellular permeability, and alter goblet cells 58-62. Chronic alcohol increases the thickness of the intestinal mucus layer in patients with alcohol use disorder 63.

M cells are associated with Peyer's patches of the small intestine, isolated lymphoid follicles, and colonic patches 64. M cells offer specialized capabilities over enterocytes in their ability to perform endocytosis and transcytosis, allowing for handling a wide and varied range of microbial products 64, 65. M cells act to transport antigens to both macrophages and LP-DCs and are consequential in antigen monitoring for the intestinal immune response 64, 66. M cells have a unique structure in the basolateral pocket which contains a partner B lymphocyte that helps with M cell maturation 64. M cells can also be induced by cytokines such as the receptor activator of nuclear factor-kappa-Β ligand (RANKL) 65. In Peyer's patches, antigens transported through M cells are exposed to DCs. Subsequently, DCs present the antigens to T and B cells leading to the determination of the immune response 67, 68. Tumor necrosis factor receptor 2 (TNFR2) is also required for stimulation of M cells. Stimulation of M cells triggers transcytosis of microbes into the lamina propria further exacerbating inflammation and disease progression 69. Impacts of ALD on M cells have been documented in mice fed a 45-day ethanol diet. Ethanol abuse induced structural changes in M cells such as mitochondrial swelling and disruption of cell-matrix within the cells 70. Changes in wild type mice fed a 19-week ethanol Lieber-DeCarli diet found an overall decrease in cells within Peyer patches and particularly fewer T and B cells 71. Overall functional changes in M cells during ALD are currently understudied. Given the transcytosis functionality of M cells, research into M cell function during ALD could offer further detail into their role in the intestinal immune system and microbiota interaction.

Enteroendocrine cells (EECs) are a very small part of the epithelium, but their function is far-reaching within areas such as the small intestine, colon, and gut microbiota. In response to nutrients, EECs secrete peptide hormones like somatostatin, motilin, cholecystokinin, neurotensin, vasoactive intestinal peptide, enteroglucagon, gastric inhibitory peptide (GIP), glucagon-like peptide-1, peptide YY, oxyntomodulin and glucagon-like peptide-2, histamine, and secretin to control gastrointestinal function and can also release cytokines in response to microbial metabolites and pathogenic molecules 72. Short-chain fatty acids (SCFAs) are detected through G protein receptors 41 and 43 (GPR41, GPR43) expressed in EEC and promote inflammatory responses in mice 72-74. These activated cytokines can alter the gut barriers through tight junction alteration 75. During inflammation, the assistance that EECs offer in the way of intestinal immune system activation comes at a cost as EECs are responsible for appetite regulation and energy metabolism 73-75. Chronic alcoholics presented a slightly significant increase in the numbers of glucagon and GIP suggesting that chronic alcohol consumption may have an effect on some endocrine cell types in the duodenal mucosa 76. It has also been reported and impairment of intestinal somatostatin production by chronic ethanol exposure in rats 77.

Tuft cells, similar to EECs constitute a very small number of cells within the epithelium and are also found throughout the colon and small intestine; however, their relative number is higher is the distal portion of the small intestine 78. Tuft cells function as a communicator amongst the intestinal immune cells. They produce the cytokine interleukin-25 (IL-25) which activates T helper 2 (Th2) cells in type 2 immune system response 79. Tuft cells proliferate in mice in response to inflammation and immune system activation 78.

**Figure 1:**
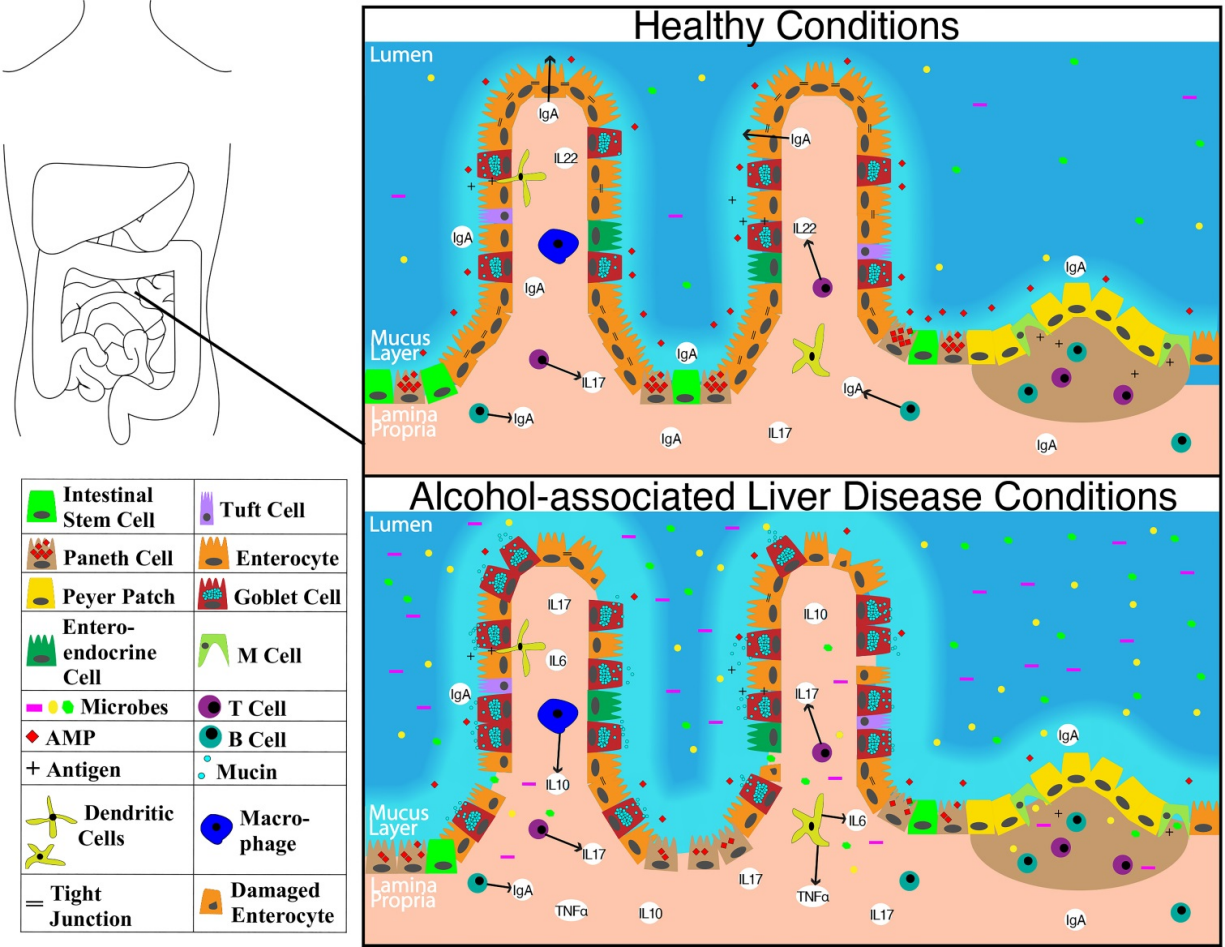
Schematic showing molecular level changes between the small intestine in healthy condition versus alcohol-associated liver disease condition. Abbreviations: AMP: anti-microbial peptide; IgA: immunoglobulin A; IL-10: interleukin 10; IL-17: interleukin 17; IL-22: interleukin 22; M cell: microfold cell; TNFα: tumor necrosis factor-alpha.

**Figure 2:**
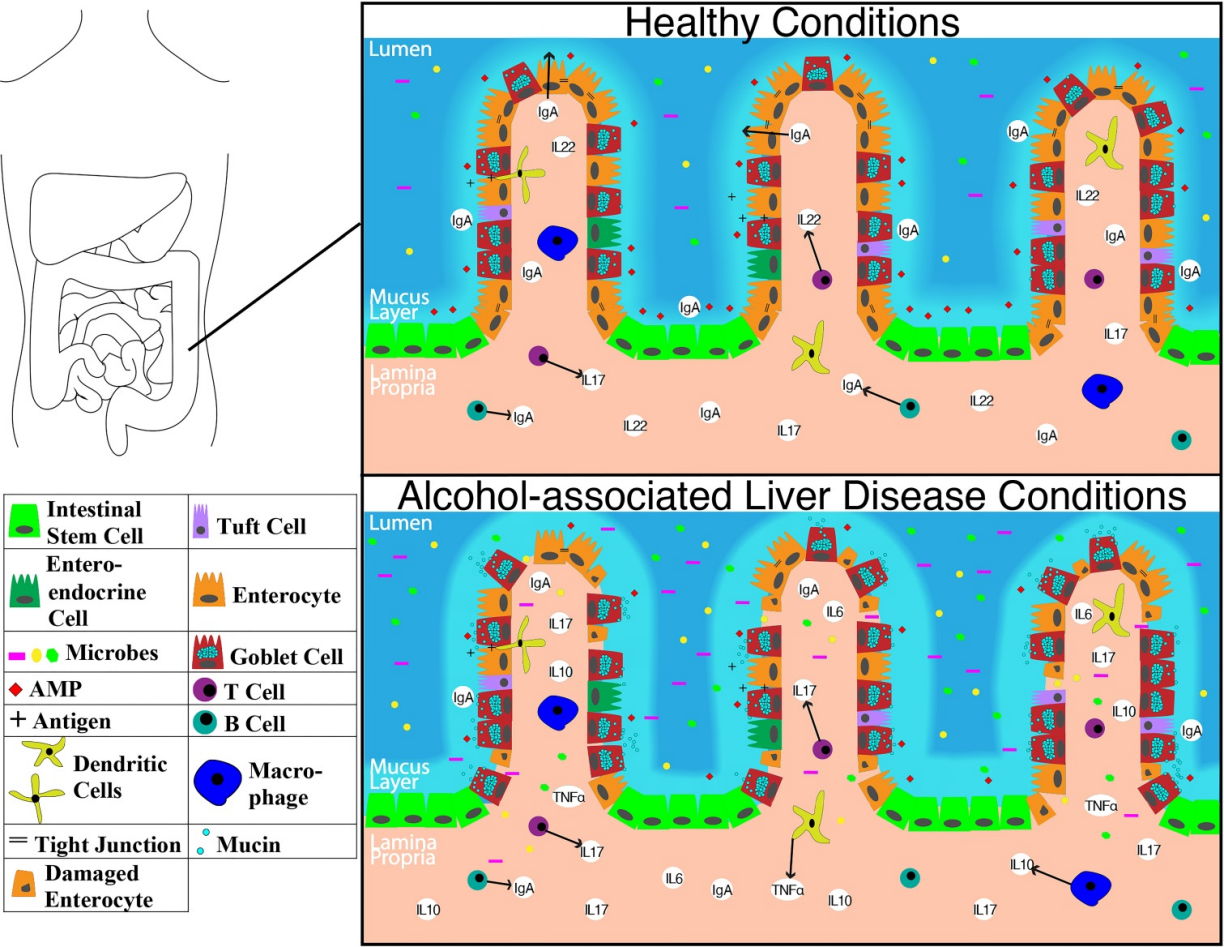
Representation of molecular level changes between the colon in healthy condition alcohol-associated liver disease condition. Abbreviations: AMP: anti-microbial peptide; IgA: immunoglobulin A; IL-10: interleukin 10; IL-17: interleukin 17; IL-22: interleukin 22; TNFα: tumor necrosis factor-alpha.

The overall structure of the small intestine with changes on a cellular and molecular level in the context of ALD is illustrated in **Figure 1.** The impact of alcohol abuse on the colon is illustrated in **Figure 2.** Major cells in the intestinal epithelium along with functions and associations with ALD are summarized in **Table 1.**

## The lamina propria and maintenance of the intestinal homeostasis

The lamina propria is another major physical barrier underneath of the intestinal epithelial cells consisting of noncellular connective tissue elements (collagen and elastin), myofibroblasts, and diverse immune cells such as CD4+ T lymphocytes, NKT lymphocytes, IgA plasma cells, B lymphocytes, innate lymphoid cells (ILCs), macrophages, mast cells, and DCs 80-85. The lamina propria contains also vascular elements such as large capillary loops, lymphatic vessels, nerves, and nerve endings 86.

LP-DCs within the lamina propria are classified based on their expression. DCs are defined as CD11c^high^, major histocompatibility class (MHC)II^high^ 87. The main subgroups of LP-DCs include those that are CD103+ CD11b+, CD103+ CD11b-, CD103- CD11b+ and CD103- CD11b- 87, 88. CD103+ CD11b+ LP DCs are CX3CR1- and can extend their dendrites outward to capture bacteria in the lumen and later present antigens to the T cells in the mesenteric lymph nodes 89. CD103+ CD11b+ LP DCs can also initiate the intestinal immune system adaptive response via recognition of flagellin through TLR5 90. TLR5 identification of flagellin prompts the production of IL-23 causing a cascading effect of ILC3 production of IL-22 and AMPs for defense against bacteria 90. During inflammation, TLRs such as TLR4 activate CD103^+^ CD11b^+^ DCs causing secretion of IL-6 which, in turn, induce T helper cells (Th) 17 differentiation 91. However, CD103+ CD11b+ DCs have characteristic tolerogenic properties, induce IgA, regulate gut-specific homing on lymphocytes 92, and stimulate the differentiation to regulatory T (Treg) cells 93. Th17 and forkhead box P3 (FOXP3)+ Tregs share developmental properties. They can transdifferentiate in response to different cytokines 94. The activation of the transcription factor FoxP3 in naïve T cells by TGF-β promotes Treg differentiation while IL6 inhibits FOXP3 95. Tolerance is crucial towards preventing autoimmunity as well as depleting commensal bacteria of the gut. Through the training of the DCs to selectively destroy harmful pathogens, a balanced immunity versus tolerance allows for proper gut function with the microbiome. CD103+ CD11b+ tolerogenic DCs help to regulate intestinal immune tolerance through promoting CD4+ T cell differentiation into Tregs 96. Notably, CD103+ CD11b+ DCs play the most prominent role in the communication with GAPs in the small intestine and the majority of antigens are delivered almost specifically to this subset 97. This subset of LP-DCs induce specific adaptive immune responses that control the intestinal homeostasis 98. Of note, this subset is rare in the colon where GAPs are closed. CD103+ CX_3_CR1- DCs produce retinoic acid and TGF-ß to stop any unnecessary inflammation 99.

The CD103+ CD11b- subset of LP-DCs is similar to CD8+ DCs in lymphoid organs 100, 101. They can migrate in a CCR7 dependent manner to the mesenteric lymph nodes (MLN) to present luminal antigens to T cells 101. These cells are more predominant in lymphoid tissues, Peyer's patches, and also in intestinal draining lymph nodes such as MLNs 102. Differentiation of CD8+ effector T cells under inflammatory condition seems to be dependent on these cells that migrate into the lymph 101.

The CD103- CD11b+ DCs subset is closely related to macrophages, is always found in the lamina propria, and activate nearby immune cells by releasing inflammatory cytokines 102. CD103-CX_3_CR1+ DCs promote inflammation through IL-6 and tumor necrosis factor-alpha (TNFα) 97, 103. Furthermore, this subset stimulates IFN-γ and IL-17 producing effector cells after lymphadenectomy 104. It has also been reported that CD103^+^ CD11b^-^ and CD103- CD11b+ cDCs can drive Th1 differentiation rather than the CD103^+^ CD11b^+^ DCs 104-106. IL-10 production from monocyte-derived DC and macrophages has also been reported to diminish inflammatory responses in the intestinal lamina propria 107. CX_3_CR1+ LP DCs can extend their dendrites to receive and monitor antigens from the lumen 89. Along with capturing, CX_3_CR1 also allows for proper transportation of these antigens to other DCs, as mice without CX_3_CR1 have reduced capability of pathogen uptake and transportation from DCs to areas such as the mesenteric lymph nodes 108. Information on the CD103- CD11b- subset which is believed to have originated from Peyer's patches or SILT is currently limited aside from their transport of luminal antigens via afferent lymphatics to the mesenteric lymph nodes 104.

Uncovering the structure and function of LP-DCs has offered insight into one of the crucial components of the intestinal immune systems adaptive response. Antigen-specific T cell responses and accessory cell function of myeloid DCs is greatly strained in human blood by alcohol drinking 109. In Rhesus macaques and mice, alcohol-reduced the number of DC pools found in the bone marrow and a noted reduction in the ability to initiate T-cell development 110. Despite this information, the effect of alcohol abuse on LP-DCs remains to be characterized.

ILCs are also present in the lamina propria and include ILC1s, ILC2s, and ILC3s 4. ILC1s are distinctive in their production of IFN-γ and found at mucosal inflammation locations while ILC2s are noted for their response to parasitic infections 4. ILC3s are the focal group when referencing the intestinal immune system due to the interleukins it produces and the impacts on the gut. RAR-related orphan receptor gamma (RORγt) is required by ILC3s for proper function 111. ILC3s can generate interleukins IL-17 and IL-22, each causing a cascading innate immune reaction in the intestinal system. IL-22 production in ILC3s is stimulated by CD103+ dendritic cells after TLR5 activation and release of IL-23 112. This IL-22 production has also been linked to gut microbe influenced epithelial fucosylation along with REG3γ and REG3ß expression 113-115. IL-17 accumulation causes a corresponding increase in neutrophil chemokines and a proinflammatory response, whereas IL-22 buildup offers a regenerative response 114. In the lamina propria, IL-22 production is impaired in mice with ethanol-induced liver disease, thus compromising the epithelial barrier in the gut, decreasing the production of REG3, and compounding issues such as increased intestinal permeability and bacterial translocation 116, 117.

Intestinal macrophages throughout the lamina propria have similarities in the membrane gene expression profile to macrophages in other tissues as well as monocytes, however, several key differences differentiate intestinal macrophages from macrophages of other tissues 118, 119. Intestinal macrophages do not have membrane expression of CD14 or CD89 and produce markedly fewer cytokines in response to lipopolysaccharide (LPS) 119. Intestinal macrophages can also secrete anti-inflammatory cytokines such as IL-10 120, 121. IL-10 acts together with TGF-ß to promote Treg differentiation 122. Chronic alcohol exposure increased the frequencies of CD64+F4/80+CD11b+ macrophage in the lamina propria 123. Further studies regarding the impact of alcohol abuse on intestinal macrophages are needed to understand the functionality and role during ALD.

CD4+ T cells are another important component of immune cells within the lamina propria. Th1 cells, a subtype of CD4+ T cells are noted for controlling immune responses responsible for killing intracellular parasites and perpetuating autoimmune responses through inflammatory cell activation via IFN-γ and for T and B cell growth through IL-2 interaction with T and B cells 124-126. Th2 cells producing IL-4, IL-5, IL-13 and also IL-10 are another subtype of CD4+ T cells. Th2 cells have noted involvement in allergy and humoral immune responses 127. Th1 and Th2 cells induce chronic intestinal inflammation 128, 129. IL-4 can create a healthy balance among the Th1 and Th2 cells due to its anti-inflammation properties and ability to regulate T and B cell growth, contrary to IL-2 129, 130. IL-10, as previously discussed also can have anti-inflammatory properties and can help to abate the production of IL-2 and IFN-γ 131. Other Th2 cytokines such as IL-5 assist with epithelial cell growth in the colon during inflammation through activation of macrophages 132. IL-13 expression, on the other hand, has been linked to higher incidences of apoptosis in intestinal epithelial cells during inflammation 132. Exacerbation of Th2 responses neutralizes the Th1 mediated microbial action. Thus, a balanced Th1 and Th2 response is required to maintain homeostasis. During ethanol-induced liver disease in mice, higher levels of a Th1 cytokine, IL-12, were increased in the liver, serum, and lungs stimulating IFN-γ production 133. However other studies found that alcohol consumption decreases IFN-γ and IL-12 in lymph nodes and spleen 134, 135 while the expression of Th2 cytokines is stable or augmented 134, 135. Therefore, ethanol drinking could be related to an immune shift toward Th2 cytokines that could contribute to damage to the intestinal immune system and the liver.

Another type of CD4+ T cell, Th17, plays a key role in inflammation. Th17 cells, along with Paneth cells in the intestine produce IL-17 which was previously discussed as a pro-inflammatory cytokine 47, 136, 137. Th17 responses can be induced by CD103+ DCs through the expression of TLR5 90. Based on a mouse NIAAA model of AH 138, IL-17 is increased in the proximal small intestine during ethanol feeding stemming from the increase in Paneth cells 47. In a study using RORγt-deficient mice, sphingosine kinase-deficient mice, and local gut anti-inflammatory, 5-aminosalicyclic acid (5-ASA)-treated mice were used to abrogate Th17 cells. These mice experienced less intestinal inflammation and less ethanol-induced liver disease 139. However, it remains unknown what would be the specific trigger of Th17 regulation locally in the intestine. In the same line, it is well known that hepatic IL-17 is induced after chronic alcohol abuse in humans and mice 136, 140.

IgA was previously discussed due to its importance in controlling bacteria after being transported to the lumen with the assistance of pIgR. IgA production in the intestine is markedly higher than other areas in the body, with two subclasses IgA1 and IgA2 existing in humans 141. IgA2 has been found to have advantages over IgA1 in the presence of bacteria due to its higher resistance to bacterial products 141. IgA1 and IgA2 are present in similar numbers within the colon lamina propria, however, IgA2 increases further down the small intestine with IgA1 found consistently throughout the small intestine lamina propria 141. Production of IgA is critical for proper maintenance of diverse gut microbiota, stopping unwarranted access of microbes, and destroying pathogenic microbes 142. In different mouse models of ethanol-induced liver disease, intestinal LP IgA-secreting plasma cells and IgA levels are reduced 143-146. In patients with severe AC, SIgA was reported reduced 147, 148. However, other studies have shown no variation in levels of jejunal SIgA secretion in patients with ALD 149 or duodenal IgA-secreting cells 148. These conflicting reports may result from subjects in different stages of the disease or different methods of IgA measurement. Future studies should be conducted to understand the causation of this variability.

Mucosa-associated invariant T (MAIT) cells also play an important role in immune response as once activated in response to bacterial infection, MAIT cells secrete cytokines such as IFN-γ, TNFα, and IL-17 to destroy bacteria-infected cells 150. During AH and AC, fewer MAIT cells are present along with a diminishing of their functional efficiency in destroying infected cells due to dysfunctional cytokine responses 150. The authors concluded that microbial products and microbiota compromise MAIT cells' antibacterial potency 150. A summary of the major cells of the lamina propria, their functions, and associations with ALD are listed in **Table 2.**

While these physical barriers and their components offer critical defense from pathogen invasion of the gut, they must also work in harmony with the microbiome for optimal health. The influence of the gut microbiome in training the immune system is a hallmark of a healthy intestinal immune system.

## Interactions of the intestinal immune system and the microbiome

The presence of a diverse set of microbes in the gut creates a mutualistic relationship with the host allowing proper gut and immune function. Intestinal microbiota gain a habitable environment and nutrients in exchange for their functions within the gut. These functions include nutrient metabolism, xenobiotic and drug metabolism, protection against pathogens, maintenance of the integrity of the mucosal barrier, and regulation at the molecular, genetic, and cellular levels of the intestinal environment which impacts the immune system 151.

The cruciality of the intestinal microbiome has long been accepted for health and proper immune response. This has been illustrated in many studies done on germ-free mice lacking microbiota. Germ-free mice have significant deficiencies in the immune system. Specifically, the lack of gut microbiome is associated with fewer intraepithelial lymphocytes, less IgA secreting plasma in the lamina propria, smaller Peyer's patches, fewer Tregs, and markedly reduced mRNA expression of AMPs from the Paneth cells such as Angiogenin-4 152-156. These deficiencies of the immune system in germ-free mice result in severe compromising of the intestine due to the inability to detect and respond to potential pathogens. Germ-free mice are more susceptible to liver fibrosis and damage in a toxic model using thioacetamide or carbon tetrachloride and in a model of acute ethanol exposure that mimics binge drinking 157, 158.

A common method of treatment in preclinical models of ethanol-induced liver disease is the use of antibiotics. The main purpose of antibiotics is to combat bacterial overgrowth and avoid translocating bacteria. The differences in antibiotics and the history of their success have led to the many different viewpoints about their current use and effectiveness in treating ALD. Antibiotics have direct impacts on the immune system within the gut due to changes in the GAPs in the colon. Colonic GAPs are typically closed to prevent bacterial translocation; however, following the administration of antibiotics, GAPs can be opened leading to a higher level of inflammation by induction of CX3CR1+ cells after recognizing higher levels of microbes and antigens 57. A potential caveat to antibiotic treatment is the development of resistance within the gut microbes. The gut microbiome resistance to antibiotics, known as the gut resistome, could occur with the long-term administration of antibiotic treatment where resistant genes transfer over amongst gut microbes including pathogens 159. This would further dampen any impact of antibiotics preventing the overgrowth of bacteria and the potential translocation of bacteria to the liver and other tissues. The result of these potential issues with antibiotics has increased the calls for a more individualized approach to prevent ALDs and alternative therapeutic options which are discussed in a later section.

A major function of gut microbes is converting dietary carbohydrates into organic acids. Lactate and SCFAs such as acetate, propionate, and butyrate can all be end-products created by the gut microbes 160. Each of these can be used for communication amongst microbes but can also be used for other purposes. Butyrate serves as an energy substrate for intestinal epithelial cells, as it allows for enhancement of the physical barrier in the gut and can increase tight junction assembly, leading to less translocation of bacteria across this barrier 161. Butyrate also can promote mucin synthesis strengthening the mucus barrier against pathogens along with reducing the expression of nuclear factor kappa-light-chain-enhancer of activated B cells (NF-κB), a transcription factor that can activate several genes to promote inflammation 162. Other SCFAs, such as acetate and propionate can slow inflammation through binding to receptors such as GPR43 163. In addition to SCFAs, gut microbes also synthesize long-chain fatty acids (LCFAs) and medium-chain fatty acids (MCFAs). In a mouse model of intragastric feeding of alcohol for 3 weeks, the synthesis of saturated LCFAs was found markedly reduced and was linked to an imbalance in gut microbes 164. The synthesis of saturated LCFAs by the microbiome is an important component of maintaining homeostasis within the gut microbiota along with maintaining a protective intestinal gut physical barrier 164.

The gut microbiota influences the expression of neutrophil chemokines through the promotion of naïve CD8+ to CD4+ T cells. In the mucosa of the small intestine, specific microbiota induces the differentiation of T-helper cells and secretion of IL-22 along with IL-17 which specifically induces the expression of neutrophil chemokines 163, 165, 166.

Gut microbiota can also influence DCs through Fut2 mediated α1,2-fucosylation. Certain bacteria can produce energy from fucose metabolism. This fucosylation has been found in the intestinal epithelial cells to increase during acute inflammation which causes the DCs to produce IL-23 167. IL-23 then triggers IL-22 production of RORγT+ ILCs 167. This fucosylation also has been found to help with pathogenic microbes by reducing their virulent genetic expression and improving the tolerance of the host to pathogens 167. Fucosylation is linked to bacteria located within the gut as studies on germ-free mice found no gut fucosylation after weaning pups from their mother 31. Bacteria such as segmented filamentous bacteria have been linked to increases in fucosylation in the intestine, thus impacting the production of cytokines and immune response in the host 31.

Gut microbes can also provide influential benefits on gene-level expression in the intestine. An example of this is the regulation of the immune response via regulation of the retinoic acid receptor alpha (RARα) 168. Retinaldehyde dehydrogenase (RALDH) enzymes form retinoic acid (RA) which is produced by DCs, macrophages, epithelial cells, and stromal cells. RA is consequential for Treg cell differentiation and reciprocal inhibition of Th17 cell development 169, 170. RALDH enzymes are expressed by DCs and macrophages along the intestine and its expression decreases during a high dose of chronic *T. muris* infection invading pathogens to fight the infection 171. In addition, bacteria from *Clostridia* class modulates the levels of RA in the gut by reducing the expression of retinal dehydrogenase 7 (*Rdh7*) in intestinal epithelial cells and consequently disrupting the RA signaling immune cells, reducing the levels of IL-22 and altering antimicrobial responses which in turn protects the commensal microbiota 172. These functions show the importance of RA signaling in intestinal immune system regulation. RA deficiency is associated with colitis-associated colon cancer and inflammatory bowel disease (IBD) 173. Alcohol abuse has been linked with the reduction of RA in the liver while it increases the levels in extrahepatic tissues including the colon 174, 175. However, the implications of the intestinal reduction of RA during ALD remain to be studied.

Segmented filamentous bacteria of the microbiota lead to the induction of Th17 cells through the stimulation of reactive oxygen species (ROS) enhancing IL-1β secretion, IL-6, and TGF-β 176, 177. Th17 cells are a subset of T helper cells that produce IL-17A. IL-17A stimulates epithelial cells to release pro-inflammatory factors such as C-X-C Motif Chemokine Ligand 1 (CXCL1) and C-C Motif Chemokine Ligand 20 (CCL20), allowing recruitment of immune cells to areas of inflammation and can promote tissue damage 178, 179. Other proinflammatory cytokines such as IL-6 can drive the development of Th17 cells while stymying Tregs 180. The response of Th17 cells against harmful pathogens is critical and requires a fine balance as too strong of response can lead to damaging inflammatory or autoimmune diseases. The impact on Th17 cells due to ethanol-induced dysbiosis associated with ALD remains to be further characterized at this point. *Clostridia* production of SCFAs has been related to the induction of TGF-β and induction of Treg cells 181. *Bacteroides fragilis* is required for the differentiation of functional Tregs producing IL-10 expression and prevent colitis 181. *Bifidobacteria* has also been involved in Th17 differentiation along with regulating the balance of Th1 and Th2 responses and stimulation of CD8+ cells 182, 183.

The microbiota also can perform quorum sensing in the gut which regulates gene expression due to microbe cell-population density fluctuations. Microbiota creates and releases chemical signals called autoinducers that operate on a positive feedback loop with cell density 184, 185. Through sensing cell population density, bacteria can effectively communicate with one another through the autoinducers and build a coordinate response through altering gene expression to effectively help regulate immune responses in cells 186.

During ALD, disruptions occur within the microbiome that accelerates and exploit damage previously mentioned in the intestinal barriers. The most common impacts involve bacterial overgrowth within the intestine along with compositional changes 187, 188. Increased harmful microbes can penetrate the gut barriers damaging the intestine and leading to translocation of bacteria to the liver where damage may also occur 189. TLRs and other pattern recognition receptors within the gut activate an innate immune system response due to the abundance of bacteria in the gut during ALD, causing a pro-inflammatory response in human patients with severe AH 190, 191.

The collective intestinal fungi that act symbiotically with the host known as the intestinal mycobiome contributes to the immune system and also undergo major impacts in disease conditions 192. C-type lectin receptors (CLRs) are instrumental in the immune response to fungi and include such receptors as dectin 1 and dectin 2 193, 194. In mice, dectin 1 amplifies cytokine production and phagocytosis in response to fungal overgrowth 193. In mice and alcohol-dependent patients, a long-term ethanol-containing Lieber-DeCarli liquid diet has been associated with the overgrowth of *Candida albicans* (*C. albicans*) causing a decrease in intestinal fungal diversity 192, 195. Levels of the toxin candidalysin, which is a peptide toxin produced by *C. albicans* were associated with progression of ethanol-induced liver disease in mice 195. Studies into the role of dectin 2 have shown its importance for immune system response to dysbiosis of the mycobiome through inducing Th17 production 194. The inflammatory cytokine produced by Th17 cells and neutrophils, IL-17 is a key factor in the immune system defense against fungal dysbiosis and overgrowth 196. During AH and AC, the overgrowth of *C. albicans* and the resulting pro-inflammatory and immune response contributes to increased intestinal permeability further exacerbating liver damage and was associated with increased mortality in patients with AH, and increased fecal levels of candidalysin which were also associated with higher mortality 192, 197.

## Interactions of the microbiome and the liver immune system

**Figure 3:**
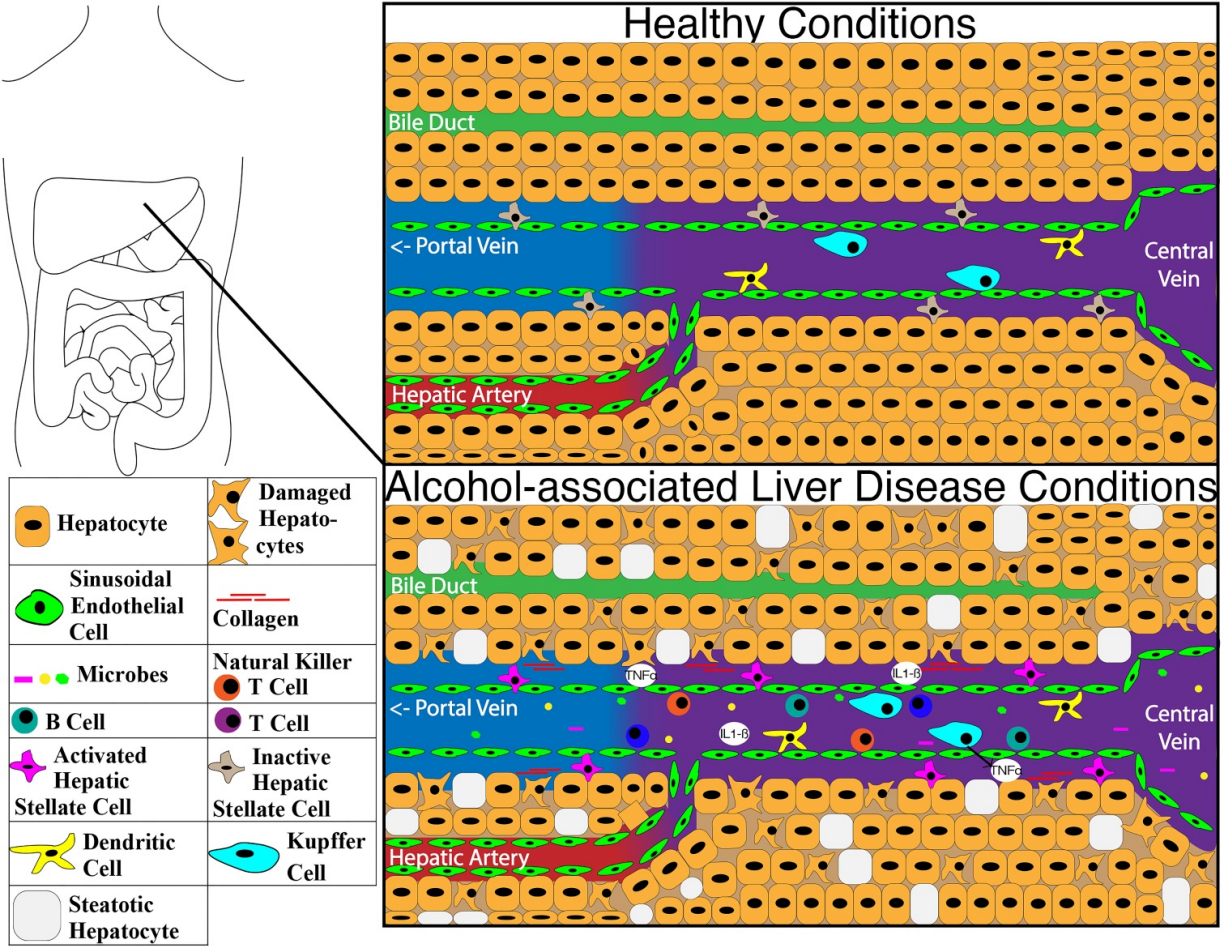
Image of differences within the liver between healthy condition and alcohol-associated liver disease. Abbreviations: IL1-ß: interleukin 1 beta; TNFα: tumor necrosis factor-alpha.

The liver immune system has limited interaction with the microbiome under normal healthy conditions. With the main connection points between the liver and microbiome being only through bile ducts and the portal vein, only select substances can get through the intestinal barrier and move into the liver. This is illustrated in **Figure 3** with the structure of the liver under healthy conditions and the impacts of ALD changes on the molecular structure. Dendritic cells in the intestine can sample and transport some bacteria to the mesenteric lymph nodes through afferent lymphatics, allowing a more localized immune response 198, 199. The vessels of the lymphatic system proliferate during chronic liver disease due to an increase in the expression of *CCL21* 200. Prolonged inflammation alters the functionality of the lymphatic system, compromising even further the liver immune system 200. A major protection mechanism of the liver for the host is through secreting IgA in the bile which attaches to bacteria 201.

Disturbances within the host can push the liver immune system to act further in regulating the microbiome to protect itself from a compromised gut barrier. Dysbiosis in patients with AH has been related to overgrowth of *E. faecalis*, which is linked to a higher incidence of liver damage and mortality due to the release of the pore-forming toxin cytolysin 202. Proton pump inhibitors (PPIs), a commonly prescribed medication, have also been linked to promoting dysbiosis through *E. faecalis* overgrowth in mice with ethanol-induced liver disease fed a long-term ethanol diet and it is a risk factor for developing ALD in alcohol-dependent patients 203. Translocation of *E. faecalis* to the liver was associated with an induction of IL1B secretion by hepatic macrophages and Kupffer cells which further contribute to the disease 203. Bacterial translocation is a common occurrence under these conditions where viable bacteria pass from the lumen of the intestine through the barriers 201, 203. The liver immune system responds through the activation of Kupffer cells via receptors such as TLR4 204, 205. Kupffer cells activate an innate immune response releasing cytokines and ROS production. The released cytokines attract immune cells such as neutrophils and monocytes to the liver to combat the invading microbes, but also promote damage in the liver as well. Recruitment of T-lymphocytes and neutrophils can occur as well as hepatic stellate cell activation, ultimately leading to collagen production and consequently fibrosis 206. Finally, invariant natural killer (iNKT) cells, commonly found in the intestine, can also migrate to the liver, during bacterial translocation induced by chronic alcohol abuse, and has been found to assist in liver cell apoptosis 207.

The liver can also have an impact on the Th17 and Treg balance in the lamina propria through derivates of the bile acid metabolite lithocholic acid (LCA). 3-oxoLCA can bind to RORγT to prevent Th17 cell proliferation, whereas isoalloLCA produces mitochondrial ROS leading to an uptick in FOXP3 expression which is a master regulator of the differentiation of Tregs 208. In mice fed Lieber DeCarli ethanol diet, farnesoid X receptor (FXR), a regulator of bile acids, lipids, and glucose metabolism, is downregulated, leading to a higher amount of bile acids being produced and secreted by the liver 209. Mice with whole-body depletion of FXR treated with a chronic plus binge ethanol feeding model were susceptible to hepatic steatosis and ethanol-induced liver disease 209-211. Experimental treatment of isoalloLCA and 3-oxoLCA to naïve CD4+ T cells isolated from FXR deficient mice, did not change FOXP3 expression when compared to the controls 208. In the 3-oxoLCA treated cells, FXR did not assist with the inhibition of Th17 cells 208.

## Alternative therapeutic approaches that modulate the intestinal homeostasis to prevent alcohol-associated liver disease

Under healthy conditions, the intestinal immune system finds a balance between proinflammatory response to pathogens and preventing the pro-inflammatory responses from causing damage to vital organs. As previously mentioned, this is regulated by the gut microbiota, crucial for the maintenance of intestinal homeostasis. However, under certain conditions, this balance can become skewed, leading to a compromised intestinal immune system. Chronic alcohol abuse causes consequential impacts on the immune system, liver, intestine, and gut microbiota. Excessive alcohol use increases the permeability of the intestine, leads to inflammation, and causes tight junction dysfunction. Intestinal bacterial overgrowth and dysbiosis is also a common effect of alcohol abuse which can lead to high levels of bacterial translocation to the liver, causing irreparable damage 50, 188. Over recent years, some therapeutic approaches have targeted cells that are consequential in the immune response and regulation of the microbiome.

Therapeutic approaches targeting ILC3s in mice have become an emerging area of research over recent years. Ethanol-induced liver disease is typically associated with impairment of ILC3s in the gut from properly producing IL-22, thus fueling bacterial translocation 117. By targeting ILC3s and therefore reducing bacterial translocation, progressive liver damage could be avoided allowing for the immune system to focus the response in the gut. One such therapeutic approach on ILC3s done in mice involves Aryl-hydrocarbon receptor-ligand (AHR) indole-3-acetic acid (IAA). This approach has been found to promote the expression of REG3γ through supplementing bacteria engineered to produce IL-22, reducing the translocation of bacteria to the liver 117. Another therapeutic approach is through the administration of IL-22 producing bacteria which was also found to reduce bacterial translocation 117. Preliminary clinical data show a correlation to the overall survival rate in patients with AH to the density of IL-22 cells from peripheral blood 212, 213. F-652 is a recombinant fusion protein of human IL-22 and immunoglobulin G2 fragment crystallizable. The first human phase I clinical trial using F-652 in healthy subjects was conducted 214 and phase II clinical trial (NCT02655510) to treat patients with AH and severe AD is now completed 215. AH patients treated with F-652 presented a significant decrease in the MELD score, total bilirubin, ALT and AST, a high rate of Lille score, and reduction of marker of inflammation in plasma 216.

The cytokine osteopontin has shown controversial results in animal studies in ethanol-induced liver disease 217-219. Osteopontin is normally increased in patients with AH, however, pushing levels of expression in hepatocytes experimentally beyond the natural increase blocked TNFα and its damaging impacts on the liver 217. However, therapies targeting inflammatory cytokines such as TNFα have been demonstrated to be ineffective and increase mortality due to infections 220, 221.

Th17 offers an intriguing option as an alternative therapy approach as their presence within the immune system fights invading pathogens through promoting inflammatory responses 222-226. These inflammatory responses, however, can be problematic during chronic events such as ALD. The pro-inflammatory positive feedback response can be harmful to the liver where inflammation causes a buildup of tissue damage 140, 227 and can also promote an autoimmune response 228 and compromise the immune system tolerance of commensal gut microbes 229. Several recent studies have shown the positive impacts of stymying a Th17 cell buildup. Halofugine, an antifibrotic drug, was found to suppress Th17 differentiation and thus lower inflammation and collagen synthesis in a model of concanavalin A-induced liver fibrosis in the rat 230. This was accomplished due to Halofugine downregulates TGF-ß signaling and other reactive cytokines, leading to less inflammation and collagen buildup 230. Similarly, as mention above mouse models with a reduced number of Th17 cells presented less intestinal inflammation and reduced ethanol-induced liver injury 139. Caution should be considered when using IL-17 targeted therapies as some studies with inflammatory bowel disease and Crohn's disease have resulted in isolated cases of exacerbation of the disease or new-onset 231-233.

*Lactobacillus rhamnosus GG* supernatant (LGG-s) therapy was also found to increase mRNA expression of tight junction proteins and reducing intestinal permeability in a mouse model of chronic-binge alcohol feeding. This therapy led to a decrease of *Escherichia coli* in the liver and an overall balance restored of Tregs, Th17s, and IL-17 234, 235. Most importantly, there is a completed, yet to be published clinical trial in phase II for acute AH patients (NCT01922895) that has studied whether *Lactobacillus rhamnosus GG* supplementation will improve their condition and intestinal complications. While some alternative therapies seek to address ALD treatment by significantly reducing bacteria levels overall within the host, prebiotic and probiotic therapies aim to alleviate gut microbe dysbiosis. Prebiotic therapies aim to restore commensal gut microbes through the introduction of dietary supplements. Studies using fructooligosaccharides (FOS) prebiotics in mice after the intragastric feeding model of continuous ethanol infusion showed a lessening of bacterial overgrowth and restoration of AMP levels such as REG3γ 50. FOS administration has been shown to increase ileal IgA secretion, B220+IgA+ cells, and pIgR expression in the small intestine and colon 236, 237 and induced immunoregulatory DC responses 238. The use of pectin in mice fed alcohol with a Lieber DeCarli diet avoided ethanol-induced downregulation of *Bacteroides* and protected from ethanol-induced liver disease 239. Administration of *Lactobacillus* species GG in rats, administration of *Akkermansia muciniphila* in mice, short-term oral supplementation with *Bifidobacterium bifidum* in preclinical studies have shown improvement of gut microbe homeostasis and reducing liver damage 240, 241. A clinical study where administration of *Lactobacillus plantarum 8PA3* to AH patients, and Lactobacillus* subtilis/Streptococcus faecium* supplementation in AH patients also have shown improvement of gut microbe homeostasis, less liver damage, and balancing immune response and cytokine production 242-244. Furthermore, probiotic administration of *Lactobacillus rhamnosus R0011* and* acidophilus R0052* in mice with ethanol-induced liver disease has also been linked to a reduction in TNFα, IL-1β TLR-4 in the liver 245.

Fecal microbiota transplantation (FMT) is a growing interest as an alternative therapy to treat conditions such as AH to improve the long-term mortality rate of affected patients. Initial findings from a limited number of studies performing FMT on patients with severe AH found higher survival rates and less toxin buildup in the bloodstream in patients receiving the transplantation versus other patients that received other treatments such as corticosteroids and pentoxifylline 246, 247. FMT data to date offers a promising option for alternative therapy for AH, however, further studies are needed to confirm these results. There is a recently completed phase 1, randomized, single-blind, interventional clinical trial (NCT03416751) that included patients with cirrhosis and alcohol use disorder to assess whether FMT improves the prognosis of these patients. The study showed that FMT is associated with lower alcohol consumption and craving which was accompanied by reduced levels of serum IL-6 and lipopolysaccharide-binding protein (LBP), an increased amount of butyrate/isobutyrate, and increased microbial diversity 248. FMT has been related to the restoration of CD4+, CD8+, and B220+ in the small intestine and CD4+ in the colon after broad-spectrum antibiotics for 8 weeks 249. The study found with those receiving an FMT a reduction in short-term alcohol use along with improvements in microbial diversity which has been inversely linked to the severity of alcohol-use related diseases 248. It would be relevant to study the impact of FMT in the LP immune system of patients with ALD and establish a deeper relationship between certain bacterial composition and the mucosal immune system.

The use of antibiotics to control ethanol-induced bacterial overgrowth and prevent ethanol-induced liver disease in rodent models has been previously demonstrated 249, 250, however, the short term use of paromomycin in patients with ALD did not result in improvement of liver function tests 251. The use of antibiotics has been related to changes in DC, memory/effector T cells, Th17, Tregs ILC3, IL-22, Th1 and Th2 in the small intestine, large intestine, spleen, and mesenteric lymph nodes in mice 249, 252, changes in immunity to vaccines in humans and it has been related with food allergies in humans 253, 254. Depletion of gram-negative bacteria compromises TLR4 and MyD88 signaling while depletion of gram-positive bacteria reduces TLR2 signaling and both effects result in a reduction of Reg3g levels 252, 255. These results indicate that despite antibiotics are a good alternative to reduce bacterial overgrowth, its use could promote drastic changes in the immune-microbiome interactions vital for health. However, some antibiotics such as rifaximin have been shown beneficial for hepatic encephalopathy in cirrhosis and had an impact on gut bacterial metabolites such as an increase in saturated and unsaturated fatty acids without significant changes in microbial abundance 256. There is a completed but yet to be published phase 3, interventional, randomized clinical trial (NCT02281929) that has proposed the use of augmentin (amoxicillin, clavulanic acid) in conjunction with oral corticotheraoy (prednisolone) to test whether it has positive effects in patients with AH. This is a very interesting approach however levels of intestinal *C. albicans* have been related to the use of certain antibiotics, among them amoxicillin 257, 258.

As previously mentioned, during ALD overgrowth of certain bacteria such a cytolytic *E. faecalis* contributes to liver damage after translocation. The presence of cytolysin in stools correlates with mortality in patients with AH. Mice gavaged with cytolytic *E. faecalis* exhibited increased levels of cytolysin in the liver and developed a more severe ethanol-induced liver injury. Cytolysin forms pores 259 and induces hepatocyte death 202. To counteract this dysbiosis, bacteriophages targeting cytolytic *E. faecalis* were found to significantly diminish cytolysin and the impacts of AH such as liver damage and hepatic cell death in mice 202. Future studies can determine if this alternative therapy can translate to humans.

The connection of the bile ducts between the liver and intestine offers a potential option to alternative therapy approaches. Restoring the homeostasis of bile acids is a necessary step to preventing further liver injury in patients with ALDs. The potential key target to restoring homeostasis within bile acids is FXR, with a study reporting positive results of reduced liver injury in rats 260. By using Dihydroartemisinin (DHA), an anti-malaria drug, FXR expression was restored suggesting FXR as a molecular target of DHA 260. In another study, ethanol-induced liver disease was reduced in mice using an intestine-restricted FXR agonist fexaramine through the reduction of IL-1ß and TNF in the liver 261. Further studies can help to uncover the mechanisms for upregulating FXR expression and the reestablishment of bile acid homeostasis. There is an interventional phase 2 completed a clinical trial that is using obeticholic acid (OCA), an FXR agonist, in moderately severe AH patients (NCT02039219). Early termination of the study did not allow researchers to collect enough samples to study changes in innate immunity. However, studies in rodent models have shown that OCA reduces the production of TNF-α by intestinal DC and their differentiation into mature DCs in models of colitis avoiding intestinal inflammation 262, 263.

Nutrition-based therapies are another targeted alternative therapeutic approach to ALD. Given the influence on diet and microbiota derived-metabolites such a SCFAs, MCFAs, and LCFAs on the host, these potential therapies could offer promising results for attenuating ALD. Supplementation of SCFAs, MCFAs, or LCFAs in both mice and rats, have all been linked to reduce liver damage and improve the intestinal gut barrier 264, 265. MCFAs and LCFAs in particular have been found to enhance the expression of intestinal tight junctions 164, 266, 267. Future research on the impacts of SCFAs, MCFAs, and LCFAs on humans with ALD can further determine both the short-term and long-term implications of nutritional therapy. In addition, the administration of SCFA facilitates *C. albicans* clearance and Tregs inductions in mice treated with an antibiotic 268. SCFAs derived from *Clostridia* are also related to the induction of TGF-β and Treg cells 181.

Other novel therapies have emerged with promising results towards potential ways of alleviating ALD. Purified hyperimmune bovine colostrum (IMM-124E) therapy has been used in phase 3 human clinical trial (NCT02473341) in severe AH patients to reduce bacterial translocation to the liver as it contains IgG anti-LPS antibodies.

Therapies using agonists and antagonists have also shown promise and are currently moving through human trial phases. Anakinra, an IL-1 receptor antagonist is currently being tested in phase 2 clinical trial (NCT04072822) in human subjects with AH. If successful, anakinra will be able to reduce TLR4-induced inflammation which depends on signaling from IL-1. Inhibition of IL-1R signally is related with reducing expression of IL-17A by Th17 cells in mice and humans 269. Another antagonist, Selonsertib (GS-4997) in combination with prednisolone, completed phase 3 clinical trial (NCT03053063) but was not found to reduce fibrosis in affected patients 270.

Antioxidant therapy clinical trial results in AH patients using such antioxidants as N-acetylcysteine (NAC) and metadoxine have shown higher survival rates short term, however, the long-term effects and overall efficacy require further study 212. A current phase 3 clinical trial (NCT03069300) to study the effect of NAC in conjunction with prednisolone in AH patients is currently ongoing. NAC treatment under LPS challenge has been associated with changes in the immune and inflammatory response 271. In addition, mucus hypersecretion is controlled by NAC as NAC functions as a mucolytic through depolymerization of the mucin, and decreasing viscosity which decreases the adhesion of bacteria 272. The benefits of mucin depletion accompanied by a possible induction of Reg3g during ethanol-induced liver disease has been discussed above 49.

Human clinical trials using regenerative methods such as the previously mentioned IL-22 therapies are also offering positive initial outcomes 212. A current clinical trial in patients with severe AH found treatment using granulocyte colony-stimulating factor (G-CSF) in phase 4 (NCT03703674). Previous clinical trial phases showed that G-CSF leads to a higher rate of survival due to improved liver regeneration through stimulation of hematopoietic stem cells present in the liver and more neutrophils in the blood 212.

## Conclusion

While many recent studies show promise in advancing treatment towards ALD, further research is needed to combat the disease. Along with targeting ILC3s, and Th17 cells functionality for alternative therapeutic approaches, the LP-associated immune system also offers other potential targets for effective therapy. Continuing research into alternative therapies to these targets offers a promising outlook to both better understanding and treating diseases impacting gut and liver health.

## Figures and Tables

**Table 1:** Major cell types found in the intestinal epithelium, their major function(s), and impacts seen in the cells during ALD

Cell type	Layer	Major function(s)	Ald impacts	References
Enterocytes	Intestinal epithelium	Cytokine secretion (APRIL, BAFF, TSLP) and response;	Reduced expression of tight junction proteins;	4-16, 18-27, 30, 31
		AMP secretion (REG3γ, REG3ß, cathelicins);	Increased permeability;	
		IgA transport (pIgR);	Reduction in expression of REG3γ and REG3ß;	
		Formation of tight junctions (occluding, claudins, Junction Adhesion Molecules);	IgA levels reduced;	
		Express Lypd8 protein to prevent invasion of flagellated microbiota;	Apoptosis of enterocytes;	
		Express fucosylated carbohydrate structures.	α1-2-fucosylation downregulated.	
Goblet cells	Intestinal epithelium	Mucin secretion to form mucus layer;	Mucin production increases in the small intestine;	46, 51-63
		Disperse AMPs (lysozyme C, α-defensins, phospholipases, cryptdins, lectins), IgA, and mucin for mucus protective layer;	Thickening of mucus layer.	
		Secrete AMPs (RELM-ß, TFF);		
		Create goblet cell associated-passages (GAPs).		
M cells	Intestinal epithelium	Transporters of antigens to macrophages and dendritic cells through endocytosis and transcytosis;	Structural changes in M cells;	64-66, 70
		Partner with B lymphocyte.	Further research needed.	
Enteroendocrine cells	Intestinal epithelium	Help with gastrointestinal process through release of peptide hormones;	Increase in the numbers of glucagon and gastric inhibitory peptide (GIP) cells;	72-77
		Release cytokines;	Decreased somatostatin.	
		Recognize SCFAs through GPR41, GPR43.		
Tuft cells	Intestinal epithelium	Produce cytokines (IL-25) to allow communication with intestinal immune cells	Increase in number	78, 79
Intestinal stem cells	Intestinal epithelium	Replenish themselves along all cell types in the intestinal epithelium;	Dysfunction of signaling;	32
		Maintains barrier.	Reduces replacement of cells.	
Paneth cells	Intestinal epithelium	Produce and release AMPs (lysozyme C, α-defensins, phospholipases, cryptdins, and lectins)	Increase in number in the proximal small intestine;	10, 11, 35, 40-44, 46-50
			AMP α-defensins reduced;	
			REG3 expression reduced.	

Impacts seen during ALD include evidence from human patients and/or evidence from mouse models fed various ethanol diets. Abbreviations: ALD: alcohol-associated liver disease; AMP: anti-microbial peptide; APRIL: A proliferation-inducing ligand; BAFF: B cell-activating factor of the tumor necrosis factor family; GAPs: goblet cell associated-antigen passages; GIP: gastric inhibitory peptide; GPR: G protein receptors; IgA: immunoglobulin A; Lypd8: Ly6/Plaur domain-containing 8; M cell: microfold cell; pIgR: polymeric immunoglobulin receptor; REG3: regenerating islet-derived 3; RELM- ß resistin-like molecule ß; SCFAs: Short-chain fatty acids; TFF: trefoil factor; TSLP: thymic stromal lymphopoietin.

**Table 2:** Major cell types found in the lamina propria, their major function(s), and impacts seen in the cells during ALD

Cell type	Layer	Major function(s)	ALD impacts	References
Dendritic cells	Lamina propria	Microbe sampling, seize harmful pathogens in the lumen;	Fewer pools of dendritic cells	89-93, 96, 97, 101, 102, 109, 110
		Instigate intestinal adaptive immune response through TLR5;		
		Induce IgA, stimulate Treg cell and Th17 cell differentiation;		
		Specific subsets of DC associate with GAPs;		
		Present luminal antigens to T cells within mesenteric lymph nodes inducing tolerance or immunity.		
ILC3s	Lamina propria	Interleukin production (IL-17, IL-22) in the intestine spurring an innate immune response	Interleukin production is hindered Amplifies bacteria translocation.	90, 112, 114, 116, 117
Macrophages	Lamina propria	Produce fewer cytokines in response to LPS compared to other tissue macrophages;	Increase in number	119-122
		Secrete anti-inflammatory cytokines (IL-10) that can assist with differentiation of Tregs.		
Th1 and Th2 cells	Lamina propria	In balance, assist with immune response through inflammation and regulate T and B cell proliferation.	Balance is compromised, favoring Th2 cells and promoting tissue damage.	129, 130, 133-135
Th17 cells	Lamina propria	Key player in inflammation;	Increase in number;	47, 90, 136, 137, 139, 140
		Activated by lamina propria dendritic cells.	Pro-inflammatory.	
IgA	Lamina propria	Prevents invading microbes from gaining access through barrier;	Conflicting findings, some have reported overall decrease and other findings have shown no change;	142-149
		Destroys pathogenic microbes	Further research needed.	
MAIT cells	Lamina propria	Release cytokines (IFN-γ, TNFα, IL-17) Destroy cells infected with bacteria	Fewer presense and less efficient in function.	150

Impacts seen during ALD include evidence from human patients and/or evidence from mouse models fed various ethanol diets. Abbreviations: ALD: alcohol-associated liver disease; GAPs: goblet cell associated-antigen passages; IFN-γ: interferon-gamma; IgA: immunoglobulin A; IL: interleukin; ILCs: innate lymphoid cells; LPS: lipopolysaccharide; MAIT: mucosa-associated invariant T cells; Th: T helper cells; TLRs: toll-like receptors; TNF: tumor necrosis factor; Treg: regulatory T cells.

## Abbreviations

5-ASA: 5-aminosalicyclic acid
AC: alcohol-associated cirrhosis
AH: alcoholic hepatitis
ALD: alcohol-associated liver disease
AMPs: antimicrobial peptides
AHR: Aryl-hydrocarbon receptor-ligand
APRIL: A proliferation-inducing ligand
AUD: alcohol use disorder
BAFF: B cell-activating factor of the tumor necrosis factor family
*C. albicans*: *Candida albicans*
CLRs: C-type lectin receptors
CCL20: C-C Motif Chemokine Ligand 20
CXCL1: C-X-C Motif Chemokine Ligand 1
DCs: dendritic cells
DHA: Dihydroartemisinin
*E. faecalis*: *Enterococcus faecalis*
EECs: enteroendocrine cells
FMT: fecal microbiota transplantation
FOS: fructooligosaccharides
FOXP3: forkhead box P3
FXR: farnesoid X receptor
G-CSF: granulocyte colony stimulating factor
GAPs: goblet cell associated-antigen passages
GIP: gastric inhibitory peptide
gp130: glycoprotein 130
GPR: G protein receptors
IAA: indole-3-acetic acid
IBD: inflammatory bowel disease
IFN-γ: interferon-gamma
IgA: immunoglobulin A
IL: interleukin
ILCs: innate lymphoid cells
ISCs: intestinal stem cells
LCA: lithocholic acid
LCFAs: long-chain fatty acids
LGG-s: *Lactobacillus rhamnosus* GG
Lgr5: leucine-rich repeat-containing G-protein couple receptor 5
LPS: lipopolysaccharide
LP-DCs: lamina propria dendritic cells
Lypd8: Ly6/Plaur domain-containing 8
M cells: microfold cells
MAIT: mucosa-associated invariant T cells
MCFAs: medium-chain fatty acids
MHC: major histocompatibility class
MLN: mesenteric lymph nodes
NAC: N-acetylcysteine
NF-κB: nuclear factor kappa-light-chain-enhancer of activated B cells
NODs: nucleotide-binding oligomerization domain-containing proteins
OCA: obeticholic acid
pIgR: polymeric immunoglobulin receptor
PPI: Proton pump inhibitors
RA: retinoic acid
RALDH: retinaldehyde dehydrogenase
RANKL: receptor activator of nuclear factor-kappa-Β ligand
RARα: retinoic acid receptor alpha
*Rdh7*: retinal dehydrogenase 7
REG3: regenerating islet-derived 3
RELM- ß: resistin-like molecule ß
RORγt: RAR-related orphan receptor gamma
RAR: related orphan receptor gamma
ROS: reactive oxygen species
SCFAs: Short-chain fatty acids
TFF: trefoil factor
Th: T helper cells
TLRs: toll-like receptors
TNF: tumor necrosis factor
TNFR2: Tumor necrosis factor receptor 2
Treg: regulatory T cells
TSLP: (thymic stromal lymphopoietin)
VLDL: very low-density lipoprotein
